# Utilisation of QSPR ODT modelling and odour vector modelling to predict *Cannabis sativa* odour

**DOI:** 10.1371/journal.pone.0284842

**Published:** 2023-04-25

**Authors:** Kimber Wise, Nicholas Phan, Jamie Selby-Pham, Tomer Simovich, Harsharn Gill

**Affiliations:** 1 School of Science, RMIT University, Bundoora, Victoria, Australia; 2 Nutrifield, Sunshine West, Victoria, Australia; 3 Faculty of Science, Monash University, Clayton, Victoria, Australia; 4 School of Engineering, RMIT University, Melbourne, Victoria, Australia; 5 PerkinElmer Inc., Glen Waverley, Victoria, Australia; University of Brescia: Universita degli Studi di Brescia, ITALY

## Abstract

Cannabis flower odour is an important aspect of product quality as it impacts the sensory experience when administered, which can affect therapeutic outcomes in paediatric patient populations who may reject unpalatable products. However, the cannabis industry has a reputation for having products with inconsistent odour descriptions and misattributed strain names due to the costly and laborious nature of sensory testing. Herein, we evaluate the potential of using odour vector modelling for predicting the odour intensity of cannabis products. Odour vector modelling is proposed as a process for transforming routinely produced volatile profiles into odour intensity (OI) profiles which are hypothesised to be more informative to the overall product odour (sensory descriptor; SD). However, the calculation of OI requires compound odour detection thresholds (ODT), which are not available for many of the compounds present in natural volatile profiles. Accordingly, to apply the odour vector modelling process to cannabis, a QSPR statistical model was first produced to predict ODT from physicochemical properties. The model presented herein was produced by polynomial regression with 10-fold cross-validation from 1,274 median ODT values to produce a model with R^2^ = 0.6892 and a 10-fold R^2^ = 0.6484. This model was then applied to terpenes which lacked experimentally determined ODT values to facilitate vector modelling of cannabis OI profiles. Logistic regression and k-means unsupervised cluster analysis was applied to both the raw terpene data and the transformed OI profiles to predict the SD of 265 cannabis samples and the accuracy of the predictions across the two datasets was compared. Out of the 13 SD categories modelled, OI profiles performed equally well or better than the volatile profiles for 11 of the SD, and across all SD the OI data was on average 21.9% more accurate (p = 0.031). The work herein is the first example of the application of odour vector modelling to complex volatile profiles of natural products and demonstrates the utility of OI profiles for the prediction of cannabis odour. These findings advance both the understanding of the odour modelling process which has previously only been applied to simple mixtures, and the cannabis industry which can utilise this process for more accurate prediction of cannabis odour and thereby reduce unpleasant patient experiences.

## 1. Introduction

Cannabis sativa is an herbaceous plant that originated in Central Asia and has been utilised for its industrial [1], ornamental [2], nutritional [3], medicinal [4], and recreational [5] potentials. Odour is conferred by the detection of volatile compounds within the nasal cavity (olfactory system) during inhalation of air, or consumption of food or liquid, via the ortho- or retro-nasal routes, respectively [6, 7]. The detection and recognition of odours by the brain can illicit physiological and psychological responses, due to the close association between odour processing and memory [8, 9]. For *Cannabis sativa*—a medicinal flower with a strong odour often administered through inhalation—odour is closely associated with quality perception and can impact the pleasantness of the medicinal experience, which in turn can impact the likelihood of redosing [10]. Accurate descriptions and consistency of product odours are therefore important for medicinal cannabis patients to promote the likelihood of achieving their therapeutic goals.

Within the cannabis industry, strain diversity is expanding, which is creating new phenotypes that require the determination of odour prior to market presentation. New phenotypes can also be created accidentally, when seed-propagated (cross-fertilised) plants are presumed to maintain their parent strain traits without proper assessment, resulting in the mis-naming of strains and associated misattribution of odour characteristics [11, 12]. Accordingly, all new strains and seed-propagated plants should be phenotype-tested to avoid inaccurate descriptions of medicinal products in the market. Whilst chemical composition (including volatiles) can be determined relatively easily through established gas chromatography (G-C) analytical methods [13, 14], these are only partially informative to odour prediction [15], and therefore current methods for determination of sensory properties relies on a slow, costly, and subjective sensory assessment by panellists [16]. Accordingly, there is a need for additional tools to measure cannabis odour and better understand the connection between volatile profiles and odour perception.

Various measures have been developed to aid in the characterisation of odours, including odour detection threshold (ODT), which is the lowest concentration that 50% of participants will detect a compound [17, 18], and odour descriptors (OD), which are common odours (items) recalled from memory such as ‘floral’ or ‘balsamic’ when a compound is detected [19]. These measures are useful for describing odour perception for samples with a single compound, however, they fail to inform the perception of mixtures. Whilst olfactory signals are produced following the detection of an odorant individually and within a mixture, the processing of the olfactory signals from a mixture occurs on a batch/ profile level, which results in the perception of a single odour rather than multiple individual odours [20]. Even though individual odorants and their OD’s cannot be distinguished as part of a mixture, it is understood that an odorant’s presence contributes to the overall odour [21]. Even low concentrations of compounds—below their ODT—have been known to influence the perception of odorant mixtures [22]. Whilst natural products tend to produce highly complex volatile profiles that are uniquely perceived, volatile mixture complexity is not necessarily associated with increased odour complexity, with some highly complex mixtures exhibiting similarity known as ‘olfactory white’ [23]. Accordingly, the relationship between volatile profiles and their perception is highly complex and there is an interest to better understand how odorants interact/ add together to produce an overall single odour. Such developments would push odour-chemistry knowledge towards facilitating prediction and targeted creation of volatile mixture odours without the need for sensory analysis.

Vector addition has been proposed as a strategy for representing odorant addition within mixtures [24]. The odour activity value (OAV) describes the relative strength of an odorant in a mixture and is calculated as the ratio between its concentration and its ODT, which is then used to calculate the odour intensity (OI) of an OD for that odorant [25]. The vector model hypothesises that the OIs (per OD per compound within a mixture) are vectors in n-dimensional odour space (for n ODs), which can be added together using vector addition [24, 26]. The resulting estimate from the vector model is a profile of OI values, which represent the combination and relative strengths of ODs imparted by the mixture. The odour vector model has been successfully applied to binary [26–29], ternary [30], quaternary [31, 32], and quinary mixtures [24]. However, noting that natural product odours—such as those from flowers or fruits—are mixtures of larger numbers of volatile compounds, there is an interest in expanding this modelling process to assess its utility within the context of representative profile complexity.

One of the limitations to the application of the odour vector model to high-complexity volatile mixtures, is the need for concentration and odour property data (ODT and ODs) for each chemical, as this is used for the calculation of OI. Whilst measurement of volatile concentration is well established [33], identification of ODT and ODs for uncharacterised chemicals generally requires sensory analysis, which is slow and costly. Chemical ODs have been identified for many volatiles due to their importance within the perfumery industry, and statistical modelling has recently been applied for the prediction of uncharacterised chemical ODs [34, 35]. However, ODT data is less comprehensive, and no large-scale generalised statistical models have been published. Therefore, there is a need for additional tools to measure or predict the ODT of uncharacterised chemicals in order to facilitate a vector modelling approach to complex mixtures containing these compounds.

Statistical modelling can be used to predict chemical activities or properties from their physicochemical data, which is known as quantitative structure–activity/property relationship (QSAR/QSPR) modelling. This process and relationship has been demonstrated for a range of properties including time of maximal phytochemical concentration in circulation following ingestion and inhalation [36, 37], blood-to-liver partition coefficients of volatile compounds [38], intestinal bioavailability and antioxidant activity [39], and perception of chemical odour following inhalation [34]. Generation of these QSPR models is useful, particularly for measures such as ODT which are laborious to measure and are required for OI calculation which is an input for vector modelling [40]. Previous studies have identified that there is a relationship between ODT and physicochemical properties, including carbon chain length and functional group presence [41, 42]. Furthermore, statistical modelling of ODT from physicochemical measures has been done for small data sets of chemical families including alkanes, esters, aldehydes, mercaptans, and aliphatic alcohols [43, 44]. However, no generalised model for the prediction of uncharacterised compound ODT has been presented.

The aim of this study was to develop a generalised QSPR model for the prediction of ODT, to enable OI calculation for uncharacterised compounds. Furthermore, this study explored the application of the odour vector model to the complex volatile profiles of cannabis flowers to produce OI profiles which could inform the overall sensory descriptor (SD) of the sample. The findings from this work provide researchers and the odour industry with a tool for approximating ODT without the need for sensory testing and additionally, strengthen the understanding of the utility of the odour vector model. The demonstration of the predictive capability of OI profiles over terpene profiles to cannabis SD is a step towards fast and reproducible prediction of flower odour which has implications for both the recreational and medicinal cannabis markets.

## 2. Materials and methods

**Fig 1:**
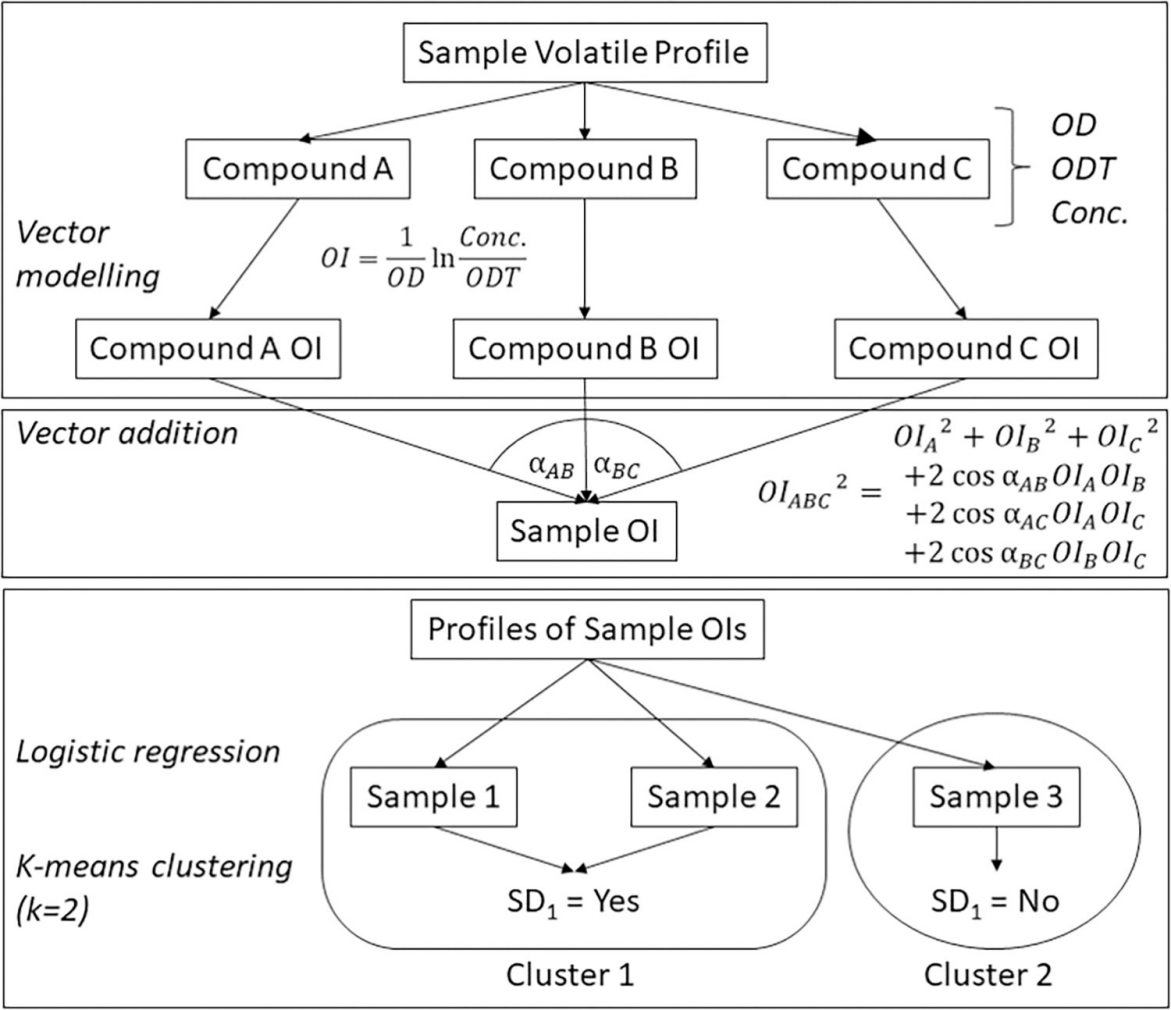
Overview of methods workflow. Cannabis flowers volatile profiles were converted into sample odour intensity (OI) profiles via vector modelling, which utilised odour descriptor (OD) data, odour detection thresholds (ODT), and compound concentrations as calculation inputs prior to vector addition. Sample OI profiles were compared to sensory data which allowed for logistic regression and k-means clustering to predict sensory descriptor assignment.

An overview of the methods utilised herein are presented in Fig 1, with further details on each method presented in their respective subsections below and detailed further in the supplemental file.

### 2.1. Sourcing of Cannabis headspace profiles

A set of 265 *Cannabis sativa* volatile terpene profiles (containing data for 18 compounds, Table S1 in S1 File) for named commercial cannabis verities were sourced from a commercial compliance testing facility (CB Labs, Novato, CA). The online database, Leafly (https://www.leafly.com/, accessed August 2022), was used to source the SDs for each named commercial variety, which were the top 3 categories listed under the ‘strain flavour’ section of each named variety’s Leafly entry. The samples were grouped into 13 SD categories (Table S2 in S1 File: Citrus, Tropical, Berry, Tree Fruit, Earthy, Mineral, Animal, Roasted, Mellow, Warming, Herbal, Floral, and Sweet), based on the location of the Leafly descriptors within a cannabis odour wheel [45]. The ODs associated with each terpene were extracted from The Good Scents Company (http://www.thegoodscentscompany.com/, accessed August 2022), as the odours listed under the ‘odor’ subsection of the ‘organoleptic properties’ section of each compound entry (Table S4 in S1 File). The ODs associated with each compound were given a numerical ranking based on the ranking of the organoleptic property for that chemical, wherein primary ranking was assigned as rank 1, secondary rank was assigned as rank 2, and so on to octonary, which was assigned as rank 8.

### 2.2. Sourcing of ODT and physicochemical measures

A set of 2,698 ODT measures of 1,274 chemicals (in water) were sourced from Van Gemert [46]. Corresponding physicochemical measures for each chemical were sourced from ChemMine Tools (https://chemminetools.ucr.edu/, accessed August 2022), which included the JoeLib descriptors: Fraction_of_rotatable_bonds, Kier_shape_2, Number_of_HBA_2, Number_of_Br_atoms, Number_of_basic_groups, Geometrical_radius, Zagreb_group_index_2, Geometrical_diameter, and Number_of_N_atoms, the OpenBabel descriptors: abonds, dbonds, MW, TPSA, and Log_P (Log_P_1), and the Smarts Search results: [#16X2H] (thiol), [$([CX3] = [CX3])] (vinylic carbon), [#6][CX3] (= O)[OX2H0][#6] (ester), [#16X2H0] (sulfide), [CX3] (= O)[OX1H0-,OX2H1] (carboxylic acid), [CX3H1] (= O)[#6] (aldehyde), and [#6][CX3] (= O)[#6] (ketone). The physicochemical measures Log P (Log_P_2) and nOHNH were sourced for each compound from Molinspiration’s online property tool kit (http://www.molinspiration.com, accessed August 2022).

### 2.3. Statistical modelling of ODT

Polynomial regression was performed in Minitab 19 statistical software package (Minitab Inc., State College, PA) to predict the natural log of the median ODT (*ln* ODT) for each of the 1,274 chemicals from their physicochemical properties listed in 2.2. Automated term inclusion was performed using the step-wise method with an alpha value of 0.05 to enter and remove, beginning with all combinations of terms up to and including 3^rd^ order. Further manual term removal was performed to achieve a model with only significant (P < 0.05) terms. Model validation was performed using the k-fold cross-validation technique, with k = 10. The accuracy of the model predictions was compared with the variations in ODT measures used to calculate the median ODT. This was done via paired t-test between the ratio of the standard deviation of the natural log of the individual ODT values for each compound with the natural log of the median ODT of each compound (ODT data variance) and the ratio of the model residual with the natural log of the median ODT (model variance), for all chemicals in the modelling-data set which were produced as a median of multiple values and had a non-zero ln *ODT* value (411 out of 1,274 chemicals).

### 2.4. Vector modelling to produce OI profiles

A vector modelling process, presented in Appendix 1 in S1 File, was adapted from Berglund [24], Yan, Liu [31], Yan, Liu [30], and Niu, Liu [26], and was applied to each cannabis terpene profile to produce a corresponding odour intensity (OI) profile. In short, the OI profile of the mixture of compounds (OIM) was generated as a 33-term vector (1 term per OD) representing the vector sum of individual compound OIs for that OD (Equation S8, Appendix 1 in S1 File). This utilised calculation of compound OAV (Equation S1, Appendix 1 in S1 File), defined as per Yan, Liu [30], wherein terpene median ODT (in water) was sourced from Van Gemert [46], except for alpha bisabolol and alpha terpinene, which were estimated using the predictive ODT model presented herein. Creation of individual compound odour profile vectors (Equation S3A, Appendix 1 in S1 File)—defined following the linear OI-lnOAV relation of individual odorants presented in Yan, Liu [31]—utilised the OD rankings presented in the GoodScents database such that OI was defined as inversely proportionate to ranking when assigned, or 0 when not assigned. The angle between compound odour profile vectors (α) was defined as the angle between vectors in 33-dimensional space due to the data covering measures across 33 ODs. For each OD, the OI of the mixture was calculated as per Berglund [24] and expanded for summation across the 18 terpenes explored per mixture (Equation S5A, Appendix 1 in S1 File).

### 2.5. Statistical modelling of cannabis sensory descriptors

Cannabis sample terpene and OI profiles were utilised separately as inputs for binary logistic regression (LR) with the logit function in Minitab 19 statistical software package (Minitab Inc., State College, PA), to predict cannabis sensory descriptors. Automated step-wise term selection was utilised with an alpha value of 0.2 to enter and remove. An initial preliminary model was created using all factors up to and including their second order terms, followed by further development into a final model with an automated selection from the same terms plus all combinations of third order terms from the factors which were selected in the initial model. The event probability (EP) outputs from LR were grouped into 2 groups using the k-means unsupervised clustering method in Minitab 19, and the group with the higher centroid was assigned the associated SD. The proportions of correct SD assignments were compared between terpene and OI profiles for an SD using 2-proportions testing with a 95% confidence interval. The overall performance of the modelling strategies using either terpene or OI data was compared by paired t-test with 95% confidence level, on the proportions of correct assignments.

## 3. Results and discussion

Whilst cannabis is known for having a strong and distinct odour, there is considerable diversity in the language used to describe cannabis odour [10]. With the recent acceptance of cannabis as a recreational drug and medicinal product, plant breeding has enabled the development of many new varieties, which often have exotic odour-inspired names and rich odour descriptions. However, cannabis naming is unregulated [47], and there is significant chemical and genetic diversity between strains of the same name [48, 49], which has resulted in a reputation within the industry of products having inaccurate descriptions or misleadingly suggestive names [50–52].

A significant demographic of medicinal cannabis users are the paediatric and geriatric patient populations, which have specific considerations for drug design and usability [53–56]. Paediatrics in particular, are sensitive to organoleptic properties such as taste and odour, with rejection occurring from unpalatable medications [57–60]. Cannabis oils are reported to have unpleasant organoleptic properties for both adults and children, which has resulted in cannabis capsule product developments [60, 61], however swallowing can be difficult in paediatrics making this unavailable to those patients [62]. For liquid oral formulations such as Epidiolex, strawberry flavouring is added [63], however no such alternative is available for raw flower products. With the inconsistencies in product labelling and descriptions, there is a risk of these special patient groups receiving medicinal products with different sensory properties to what they expect, which may result in rejection and thereby negatively affect their therapeutic outcomes. Accordingly, there is a need to increase the consistency and accuracy of cannabis labelling and descriptions to improve patient experiences and therapeutic outcomes.

The simplest measure of odour strength is OAV which is the ratio of the concentration of a compound in a sample relative to its ODT. However, OAV doesn’t inform on how that chemical will be perceived by an individual or, if present in a mixture, how other compounds are contributing to the overall odour. Odour intensity and vector addition are proposed as improved methods for determining odour perception, particularly for mixtures. As OAV is an input to OI calculation, ODT for each compound present in a mixture is required in order to perform odour vector addition of OI. Accordingly, as cannabis and other natural products contain many volatiles which have not been tested by sensory analysis to determine their ODT, there is a need to predict ODT from *in silico* measures to enable vector modelling of profiles containing these compounds.

**Fig 2:**
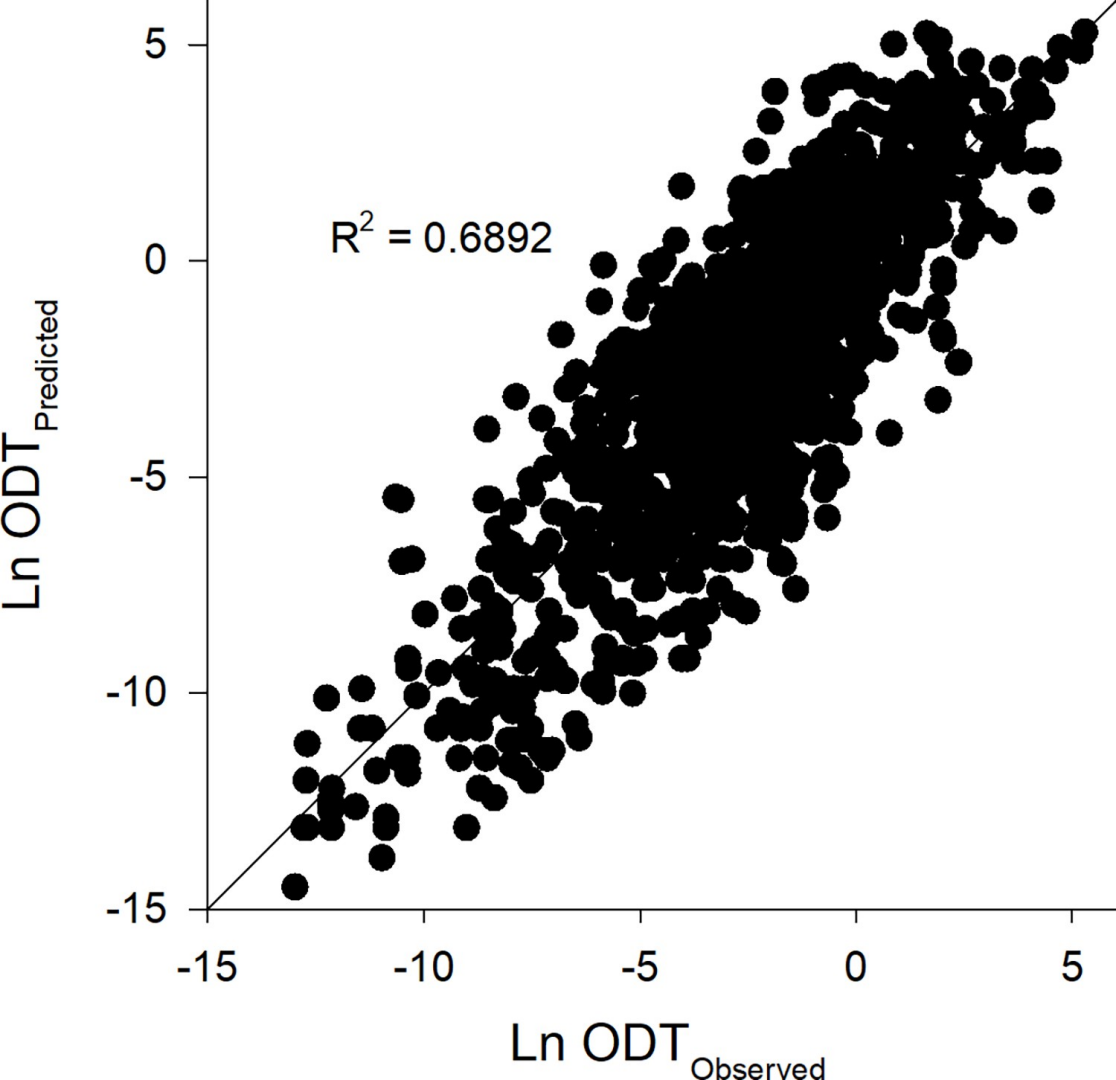
Prediction of ln ODT. Polynomial regression analysis with 10-fold cross-validation to predict ln ODT from physicochemical measures utilising median ODT values from 1,274 volatile chemicals.

Polynomial regression was utilised to generate a model for the prediction of *ln* ODT from physicochemical measures, which had an R^2^ = 0.6892 and a 10-fold R^2^ = 0.6484 (Fig 2, Table S5 in S1 File). The similarity between the two R^2^ measures indicates comparable accuracy for both the training and the test sets across the fold splits, supporting that the model is not overfit [64]. The model includes 69 total 1^st^, 2^nd^, and 3^rd^ order terms (Table S5 in S1 File), which are all significant (P < 0.05). Of these 69 terms, 49 include terms relating to the presence of functional groups, and 23 contain terms relating to molecular size. The inclusion of these properties for the prediction of ODT is consistent with previous studies which identified that ODT is impacted by the related measures of carbon chain length and functional group presence [41, 42, 65]. The model also included terms with measures relating to molecular properties (e.g. Log P), and types of bonds present (e.g. rotatable bonds), which may indicate their association with ODT perception and could therefore be the targets for further studies on physicochemical properties impacting ODT.

Previous studies have presented predictive models for ODT, however, these tend to utilise small data sets. Polster and Schieberle [65] present an ODT model from 44 compounds with a R^2^ of 0.89, Rodríguez, Teixeira [66] identified a model from 121 compounds with R^2^ of 0.769, Anker, Jurs [67] and Junkes, Arruda [68] present models from 49 compounds with R^2^ of 0.863 and 0.765, respectively, whilst Pal, Mitra [44] utilised 42 compounds to develop a model with R^2^ of 0.809 and an R^2^ of 0.813 when applied to a test set of 11 compounds. Abraham, Sánchez-Moreno [43] also modelled a log transformation of ODT against two datasets containing 206 and 353 compounds to produce models with R^2^ of 0.748 and 0.759, respectively. These works support the use of modelling of ODT from chemical properties, however, the data sets utilised in these previous models are small compared to the 10,000 identified volatile odour chemicals [69]. Therefore, as statistical models are limited in their application to compounds with properties similar to those within the modelling dataset, and the relatively small size of these models means they only account for a small proportion of the diversity of properties across all volatile compounds, these models are expected to have low generalisability for predicting ODT in new compounds. By contrast, the model presented herein utilised data from 1,274 chemicals, which is a substantial expansion in the breadth of chemical properties modelled (Table S6 in S1 File) and therefore has greater generalisability to new compounds. Furthermore, the accuracy of the model herein was found to be relatively similar to the accuracy of empirical determination of ODT through human participant sensory testing. The ratios for the ODT data variance and the model variance (defined in method 2.3 herein) were found to be on average 2.36 and 2.21, respectively (Table S7 and Fig S1 in S1 File), and were not significantly different (p = 0.771), indicating that the predicted values fall within a similar range of the reported ODT values to their median. Human olfactory sensitivity is known to be highly variable [70], and for ODT specifically, variations of up to 4 or 5 magnitudes are reported between studies of the same chemicals due to variations in sensory methodology [71]. Accordingly, the model herein which has achieved prediction of comparable accuracy could be considered as a suitable substitute for ODT in the absence of sensory testing.

It was hypothesised that odour vector modelling could be utilised to generate OI profiles for the highly complex volatile mixtures present in cannabis flowers, and that these profiles would be more informative to the overall odour than their associated terpene profiles. Additionally, whilst current odour vector modelling approaches utilise empirically solved values for the angles between compound OI vectors, it was hypothesised herein that a calculated estimation could be used in place of these values. Finally, the incorporation of OD ranking into OI calculation was hypothesised to account for the relative strengths of ODs and thereby improve the predictive power of the resulting OI profile.

**Fig 3:**
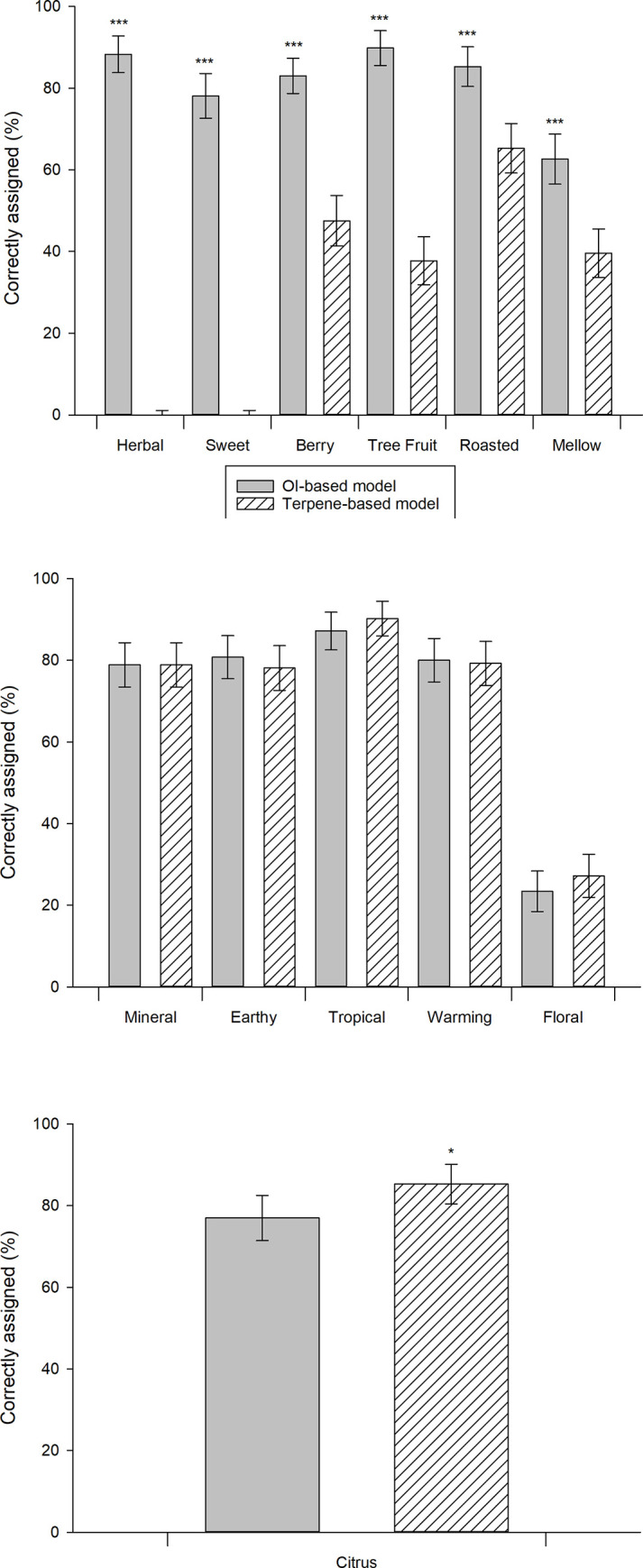
Prediction of cannabis sensory descriptors (SD) from volatile data, and OI data produced through odour vector modelling. Logistic regression analysis and k-means unsupervised cluster analysis (k = 2) to predict the SD of 265 cannabis samples. Data represent the percentage of strains correctly assigned for each SD (for each data type), with error bars representing the 95% confidence interval. A) the set of 6 SDs which were predicted significantly more accurately with OI than terpene data, B) the set of 5 SDs which were predicted with equal accuracy for both OI and terpene data, and C) the single SD which was predicted significantly more accurately with terpene than OI data. Significant differences between volatile and OI data for an individual SD are indicated by * for p < 0.05, and *** for p ≤ 0.001 calculated by Student’s t-test.

Cannabis terpene and OI profiles were explored for the prediction of cannabis SD by LR modelling. Of the 13 SD categories modelled, terpene data was able to produce models for 10 SDs (Equations S10–S19 in S1 File) whilst OI profiles could produce models for 12 SDs (Equations S20–S31 in S1 File). The performance of each model was assessed for its ability to accurately assign SDs to each sample (Fig 3). Six SD categories were able to be modelled by OI significantly (p < 0.05) better than terpene (herbal, sweet, berry, tree fruit, roasted, and mellow), 5 SD categories were modelled equally successfully for both OI and terpene data (mineral, earthy, tropical, warming, and floral), 1 SD category was modelled by terpene significantly better than OI data (citrus), and 1 SD could not be modelled by either terpene or OI data (animal). When comparing the overall performance of the two modelling strategies, it was identified that OI data produced models with an average of 70.33% accuracy and models produced from volatile data had an average of 48.39% accuracy, which was a significant (p = 0.031) difference of 21.94%.

The results indicate that odour vector modelling produces profiles which are more informative for predicting overall cannabis odour (SD) than profiles of terpene data produced by routine testing within the Californian cannabis industry. The transformation of these relatively easy-to-produce terpene profiles, into OI profiles which are more informative for odour prediction is a promising development in achieving more consistent labelling and product descriptions within the cannabis industry. Noting that these routine profiles contain only 18 volatiles of the more than 200 which contribute to the cannabis odour [72], increasing the predictive capability of these (if possible) may require considerable expansion of the breadth of chemical profiling. This increased volatile profiling would result in greater costs to manufacturers and possibly consumers and patients. Improving on the current practices is therefore the easiest and cheapest way to confer benefits to the industry and the patients. However, as some of the SDs were not able to be modelled well by either the terpene or the OI data sets (floral and animal), expansion of the volatile profiling or cannabis sample data may still be desirable to achieve more accurate modelling of these SDs. As the expansion of the volatile profiles also adds data to the corresponding OI profiles, and expansion of cannabis sample data provides more data points for the modelling, these strategies may increase the accuracy of either the terpene or the OI modelling strategies. Furthermore, the application of this strategy to explore statistical relationships between volatile and sensory profiles is not limited to the cannabis industry. Industries wherein volatile chemical profiles and sensory data are both readily collected—such as perfume or food industries—could potentially utilise this strategy to improve predictive capabilities and eventually guide alterations to volatile profiles to achieve targeted changes to sensory profiles.

## 4. Conclusion

The medicinal cannabis industry has a reputation for having inconsistent labelling and product descriptions, which contributes to a misalignment between patient sensory expectations and experiences. For special patient populations such as geriatrics and particularly paediatrics, this is of concern as they are sensitive to unpleasant sensory experiences, and this can affect their likelihood of redosing and thus achieving their therapeutic outcomes. Current routine testing procedures involve the production of volatile profiles; however, these have limited utility for the accurate determination of product odour. Accordingly, the study herein demonstrates how these routinely produced volatile profiles can be transformed through odour vector modelling to produce OI profiles which predicted cannabis SD equally well or better than terpene profiles for 11 out of 13 SD categories, equating to a significant (p = 0.031) average improvement of 21.9% accuracy. This process utilised ODT inputs which were in part produced from a novel QSPR predictive model, that was developed using an extensive dataset of 1,274 median ODT values and their physicochemical properties to achieve a regression model with a R^2^ = 0.6892 and a 10-fold R^2^ = 0.6484. Of note, these models utilised data from 18 volatile compounds, whilst cannabis odour is comprised of hundreds of compounds. As such, progressions on the work presented herein may benefit from utilisation of more comprehensive volatile profiles. Nonetheless, these findings are significant to both the cannabis industry wherein improved accuracy of odour prediction will benefit patients, as well as broader odour industries wherein expansion of the odour vector modelling process to highly complex natural odour mixtures is a substantial progression in this field with potential further applications to food products and perfumes.

## Supporting information

S1 File **Appendix 1**. Methods and equations for odour vector modelling containing Equations S1-S8, **Appendix 2**. Table S1-S8 and Fig S1, and **Appendix 3**. Equations for prediction of SD containing Equations S9-S31.
(DOCX)

## Abbreviations

G-C: gas chromatography
LR: binary logistic regression
OAV: odour activity value
OD: odour descriptor
ODT: odour detection threshold
OI: odour intensity
QSAR: quantitative structure–activity relationship
QSPR: quantitative structure–property relationship
SD: sensory descriptor
