# Supplementary material for: Utilisation of QSPR ODT modelling and odour vector modelling to predict Cannabis sativa odour
Source: PLoS One. 2023 Apr 25;18(4):e0284842. doi: 10.1371/journal.pone.0284842 (PMC10128932; doi:10.1371/journal.pone.0284842)
Supplement: S1 File — Appendix 1. Methods and equations for odour vector modelling containing Equations S1-S8, Appendix 2. Table S1-S8 and Fig S1, and Appendix 3. Equations for prediction of SD containing Equations S9-S31. (DOCX) [file pone.0284842.s001.docx]

# Appendix 1 – Methods and equations for odour vector modelling

Equation S1: Compound OAV

$${OAV}_{A}= \frac{C_{A}}{{ODT}_{A}}$$

Where, C_A_ is the concentration of compound A, and ${ODT}_{A}$ is the ODT of compound A. Where OAV is a measure of compound abundance relative to detectability.

Equation S2A: Compound OI

$${OI}_{A}= k\ln{OAV}_{A}$$

Where, *k* is a constant. Where OI is a measure of relative perceptive intensity.

Equation S2B: OD specific compound OI

$${OI}_{A,1}= \left\{ \begin{matrix} \frac{1}{{GS}_{A,1}}\ln{OAV}_{A}, for {GS}_{A,1}>0 \\ 0, for {GS}_{A,1}=0 \end{matrix} \right.$$

The OI for compound A for odour descriptor (OD) 1. Where ${GS}_{A,1}$ is the Good Scents rank of OD 1 for compound A. * note if compound A is not assigned OD 1, then ${OI}_{A,1}={GS}_{A,1}=0$.

Equation S3A: Calculating compound odour profile vectors

The odour profile vector for compound A for ODs $1-n$ (${OP}_{A,1-n}$), where each OD is thought of as existing on a perpendicular axis represented by associated eigenvectors $x_{1}-x_{n}$.

$${OP}_{A,1-n}={OI}_{A,1-n}\cdot X_{1-n}=\left( \begin{matrix} {OI}_{A,1} & \ldots& {OI}_{A,n} \end{matrix} \right)\times\left( \begin{matrix} x_{1} \\ \vdots\\ x_{n} \end{matrix} \right)=\left( {OI}_{A,1}x_{1}+\ldots+ {OI}_{A,n}x_{n} \right)$$

Equation S3B: Matrix of compound odour profile vectors

The matrix (${OM}_{A-M,1-n})$ is the odour profiles of compounds $A-M$ (${OP}_{A,1-n}-{OP}_{M,1-n}$) across ODs $1-n$.

$${OM}_{A-M,1-n}=\left( \begin{matrix} {OP}_{A,1-n} \\ \vdots\\ {OP}_{M,1-n} \end{matrix} \right)$$

Equation S4: Odour profile vector magnitude

The magnitude of the OP for a compound A is calculated as the square root of the sum of squares of eigenvector coefficients;

$$\left| {OP}_{A,1-n} \right|=\sqrt{{{OI}_{A,1}}^{2}+\ldots+{{OI}_{A,n}}^{2}}$$

Equation S5A: Odour profile direction eigenvector

The odour profile eigenvector $\bar{{OP}_{A,1-n}}$ for compound A across ODs $1-n$, is the division of the odour profile vector by its magnitude.

$$\bar{{OP}_{A,1-n}}=\frac{1}{\left| {OP}_{A,1-n} \right|}{OP}_{A,1-n}=\left( \frac{{OI}_{A,1}x_{1}}{\left| {OP}_{A,1-n} \right|}+\ldots+\frac{{OI}_{A,n}x_{n}}{\left| {OP}_{A,1-n} \right|} \right)$$

Equation S5B: Matrix of odour profile eigenvectors

The matrix of eigenvectors $\bar{{OM}_{A-M,1-n}}$ for compound $A-M$ across ODs $1-n$, is presented as;

$$\bar{{OM}_{A-M,1-n}}=\left( \begin{matrix} \frac{{OI}_{A,1}}{\left| {OP}_{A,1-n} \right|} & \cdots& \frac{{OI}_{A,n}}{\left| {OP}_{A,1-n} \right|} \\ \vdots& \ddots& \vdots\\ \frac{{OI}_{M,1}}{\left| {OP}_{M,1-n} \right|} & \cdots& \frac{{OI}_{M,n}}{\left| {OP}_{M,1-n} \right|} \end{matrix} \right)\times\left( \begin{matrix} x_{1} \\ \vdots\\ x_{n} \end{matrix} \right)=\left( \begin{matrix} \frac{{OI}_{A,1}x_{1}}{\left| {OP}_{A,1-n} \right|}+\ldots+\frac{{OI}_{A,n}x_{n}}{\left| {OP}_{A,1-n} \right|} \\ \vdots\\ \frac{{OI}_{M,1}x_{1}}{\left| {OP}_{M,1-n} \right|}+\ldots+\frac{{OI}_{M,n}x_{n}}{\left| {OP}_{M,1-n} \right|} \end{matrix} \right)$$

Equation S6: Calculate the angle between compound odour profile vectors

The angles matrix ($A$), for compounds $A-M$ across ODs $1-n$, which represents the angles between compounds in n odour space, is calculated as the cross product of the eigenvector coefficients and their matrix transposed.

$$A=\left( \begin{matrix} \frac{{OI}_{A,1}}{\left| {OP}_{A,1-n} \right|} & \cdots& \frac{{OI}_{A,n}}{\left| {OP}_{A,1-n} \right|} \\ \vdots& \ddots& \vdots\\ \frac{{OI}_{M,1}}{\left| {OP}_{M,1-n} \right|} & \cdots& \frac{{OI}_{M,n}}{\left| {OP}_{M,1-n} \right|} \end{matrix} \right)\times\left( \begin{matrix} \frac{{OI}_{A,1}}{\left| {OP}_{A,1-n} \right|} & \cdots& \frac{{OI}_{M,1}}{\left| {OP}_{M,1-n} \right|} \\ \vdots& \ddots& \vdots\\ \frac{{OI}_{A,n}}{\left| {OP}_{A,1-n} \right|} & \cdots& \frac{{OI}_{M,n}}{\left| {OP}_{M,1-n} \right|} \end{matrix} \right)=\left( \begin{matrix} \cos(\alpha_{A,A}) & \cdots& \cos(\alpha_{A,M}) \\ \vdots& \ddots& \vdots\\ \cos(\alpha_{M,A}) & \cdots& \cos(\alpha_{M,M}) \end{matrix} \right)$$

Where, $\alpha_{A,M}$, is the angle between the vectors ${OP}_{A,1-n}$ and ${OP}_{M,1-n}$, and $\cos(\alpha_{A,A})=\cos(0)=1$

Equation S7: Scalar products of odour profile vectors

The overall OI of the mixture ($OIM$) of compounds $A-M$ for OD p (${OD}_{p}$) is defined by vector addition as:

$${OIM}_{A-M,p}=\sqrt{\sum_{i=A}^{M} {{OI}_{i,p}}^{2}+\sum_{i=A}^{M-1} \sum_{j=A+1}^{M} 2\cos\left( \alpha_{i,j} \right){OI}_{i,p}{OI}_{j,p}}$$

Equation S8: Matrix of OI values for a mixture of compounds

For a mixture of compounds $A-M$ for ODs $1-n$ the OI profile of the mixture ($OIM$) is defined as:

$${OIM}_{A-M,1-n}=\left( \begin{matrix} {OIM}_{A-M,1} & \cdots& {OIM}_{A-M,n} \end{matrix} \right)$$

# Appendix 2

Table S1. Descriptive statistics of cannabis flower volatile terpene dataset used herein.

| Terpene | n | Mean | Min | Max |
| --- | --- | --- | --- | --- |
| Alpha Pinene | 265 | 0.08355 | 0.00000 | 1.13000 |
| Camphene | 265 | 0.007925 | 0.000000 | 0.060000 |
| Gamma Terpinene | 265 | 0.001698 | 0.000000 | 0.060000 |
| Carophyllene Oxide | 265 | 0.000038 | 0.000000 | 0.010000 |
| Beta Myrcene | 265 | 0.2505 | 0.0000 | 1.5700 |
| Alpha Humelene | 265 | 0.08898 | 0.00000 | 0.63000 |
| Carene | 265 | 0.000868 | 0.000000 | 0.090000 |
| Eucalyptol | 265 | 0.000453 | 0.000000 | 0.010000 |
| Alpha Terpinene | 265 | 0.000264 | 0.000000 | 0.040000 |
| Beta Ocimene | 265 | 0.1729 | 0.0000 | 2.0100 |
| Trans Ocimene | 265 | 0.03751 | 0.00000 | 0.71000 |
| Beta Limonene | 265 | 0.3302 | 0.0000 | 1.2800 |
| Terpinolene | 265 | 0.0537 | 0.0000 | 1.7300 |
| Linalool | 265 | 0.11966 | 0.00000 | 0.44000 |
| Geraniol | 265 | 0.000038 | 0.000000 | 0.010000 |
| cis-Nerolidol | 265 | 0.000189 | 0.000000 | 0.040000 |
| trans-Nerolidol | 265 | 0.003321 | 0.000000 | 0.060000 |
| Beta Caryophyllene | 265 | 0.4178 | 0.0000 | 1.3000 |

Table S2. Sensory descriptor categories

|  | Citrus | Tropical | Berry | Tree Fruit | Earthy | Mineral |
| --- | --- | --- | --- | --- | --- | --- |
| Leafly odours | - Citrus - Grapefruit - Lemon - Lime - Orange | - Tropical - Pineapple - Mango | - Berrry - Blueberry - Grape - Strawberry | - Tree fruit - Apple - Apricot - Peach - Pear - Plum - Fruity | - Earthy - Skunk - Pine - Woody | - Adhesive paste - Chemical - Diesel - Gas - Tar - Fuel - Petrol - Ammonia |

Table S2 – *continued*

|  | Animal | Roasted | Mellow | Warming | Herbal | Floral | Sweet |
| --- | --- | --- | --- | --- | --- | --- | --- |
| Leafly odours | - Pungent - Cheese - Blue cheese - Herbed cheese - Butter | - Chestnut - Tobacco - Nutty - Coffee | - Chocolate - Vanilla | - Tea - Pepper | - Sage - Herbal - Mint - lavender | - Violet - Flowery - Lavender | - Honey - Sweet |

Table S3. Number of cannabis varieties in each sensory descriptor category

| Sensory descriptor category | Count |
| --- | --- |
| Sweet | 86 |
| Citrus | 79 |
| Earthy | 68 |
| Warming | 65 |
| Mellow | 61 |
| Animal | 54 |
| Tree_Fruit | 51 |
| Berry | 42 |
| Mineral | 41 |
| Herbal | 40 |
| Tropical | 32 |
| Roasted | 25 |
| Floral | 21 |

Table S4. Terpene odour descriptors ranks sourced from goodscents

| Terpene | Goodscents descriptor (rank) |
| --- | --- |
| Alpha Pinene | Woody (1), Herbal (2), Fir needle (3), Camphoreous (4), Terpenic (5), Citrus (5), Spicy (6) |
| Camphene | Terpenic (1), Herbal (2), Citrus (3), Lemon (4). Tropical (5), Lime (7) |
| Gamma Terpinene | Woody (1), Spicy (2), Dry (3) |
| Carophyllene Oxide | Terpenic (1), Herbal (2), Woody (3), Peppery (4), Balsamic (5), Rose (6), Celery (7), Carrot (8) |
| Beta Myrcene | Woody (1) |
| Alpha Humelene | Herbal (1), Woody (2), Pine (3), Terpenic (4), Camphoreous (5), Earthy (6) |
| Carene | Citrus (1), Terpenic (2), Herbal (3), Pine (4), Solvent (5), Resinous (6), Phenolic (7), Cypress (8) |
| Eucalyptol | Eucalyptus (1), Herbal (1), Camphoreous (2), Medicinal (3) |
| Alpha Terpinene | Terpenic (2), Herbal (3), Lemon (3), Medicinal (5), Citrus (6), Thyme (7) |
| Beta Ocimene | Floral (1), Herbal (2) |
| Trans Ocimene | Herbal (1) |
| Beta Limonene | Terpenic (1), Herbal (2), Pine (3), Peppery (4) |
| Terpinolene | Pine (1), Citrus (2), Woody (3), Lemon peel (5) |
| Linalool | Citrus (1), Floral (2), Bois de rose (3), Green (4), Woody (5), Blueberry (6), Terpenic (7) |
| Geraniol | Floral (1), Fruity (2), Rose (3), Waxy (4), Citrus (5) |
| cis-Nerolidol | Floral (1), Green (2), Waxy (3), Citrus (4), Woody (5) |
| trans-Nerolidol | Floral (1), Green (2), Citrus (3), Woody (4), Waxy (5) |
| Beta Caryophyllene | Spicy (1), Clove (2), Woody (4), Dry (5) |

Table S5: Coefficients for terms used within regression equation for prediction of $\ln ODT$

| Term | Coefficient | P-Value |
| --- | --- | --- |
| Constant | 2.6863091183487500 | <0.001 |
| Thiol | -11.6351453114938000 | <0.001 |
| Kier_shape_2 | 0.4775161369247190 | <0.001 |
| Vinylic Carbon^2^ | -0.4135851147696500 | <0.001 |
| Ester × Zagreb_group_index_2 | 0.0194389906314805 | 0.019 |
| Ester × logP_2 | -1.5330349938392200 | <0.001 |
| Sulfide × Number_of_HBA_2 | 0.8505529630763500 | <0.001 |
| LogP_1 × Number_of_HBA_2 | -0.3068688739712370 | <0.001 |
| Fraction_of_rotatable_bonds × logP_2 | -7.4887515098204000 | 0.001 |
| Kier_shape_2^3^ | -0.0032881939308441 | <0.001 |
| Carboxylic Acid × LogP_1 × Fraction_of_rotatable_bonds | 1.0038170994496100 | <0.001 |
| Ester × Ketone × Geometrical_diameter | 0.5992028069642420 | <0.001 |
| Ester × abonds × LogP_1 | 0.1124859046651750 | <0.001 |
| Ester × Fraction_of_rotatable_bonds^2^ | 19.0235296180538000 | <0.001 |
| Ester × Fraction_of_rotatable_bonds×Geometrical_radius | -18.7789905675193000 | <0.001 |
| Ester × Fraction_of_rotatable_bonds × logP_2 | 1.9234585445688800 | <0.001 |
| Ester × Geometrical_radius × Kier_shape_2 | 0.3765984548584870 | 0.046 |
| Ester × logP_2 × nOHNH | -1.2932724916130000 | <0.001 |
| Aldehyde × abonds × logP_2 | 0.2126957615052850 | <0.001 |
| Aldehyde × Geometrical_radius^2^ | -3.0485014638533500 | <0.001 |
| Aldehyde × Kier_shape_2^2^ | -0.0720818469049376 | <0.001 |
| Aldehyde × logP_2^2^ | 0.3418003260167960 | <0.001 |
| Ketone × dbonds × Fraction_of_rotatable_bonds | -5.8268945232820800 | <0.001 |
| Ketone × Number_of_N_atoms × nOHNH | -2.7369287249506600 | 0.002 |
| Ketone × Geometrical_diameter × Kier_shape_2 | 0.0301562439781783 | 0.002 |
| Ketone × logP_2 × nOHNH | -1.5405927852002800 | <0.001 |
| Thiol × TPSA × Kier_shape_2 | 0.0423980276300025 | <0.001 |
| Thiol × Number_of_HBA_2 × Zagreb_group_index_2 | -0.0694893361069941 | <0.001 |
| Thiol × Geometrical_radius × MW | 0.0528770998272413 | 0.017 |
| Sulfide × abonds × Fraction_of_rotatable_bonds | -1.2967189808414300 | <0.001 |
| Sulfide × abonds × nOHNH | 1.0483666778711200 | <0.001 |
| Sulfide × Number_of_N_atoms × Kier_shape_2 | 0.4680346264114280 | <0.001 |
| Sulfide × Geometrical_diameter × nOHNH | -0.4240664868774620 | <0.001 |
| Sulfide × Geometrical_radius^2^ | -2.8600460109351500 | <0.001 |
| Vinylic Carbon × abonds × Number_of_N_atoms | -0.1217367577138330 | 0.003 |
| Vinylic Carbon × dbonds × Geometrical_diameter | 0.1166048402493670 | <0.001 |
| Vinylic Carbon × dbonds × Kier_shape_2 | -0.0803754581832530 | <0.001 |
| Vinylic Carbon × TPSA × nOHNH | 0.1394455585908410 | <0.001 |
| Vinylic Carbon × Number_of_HBA_2 × nOHNH | -2.5027408576355000 | <0.001 |
| Vinylic Carbon × Fraction_of_rotatable_bonds^2^ | -4.7953028713811200 | 0.001 |
| Vinylic Carbon × Fraction_of_rotatable_bonds × Kier_shape_2 | 0.3113070964584520 | 0.011 |
| abonds^2^ × LogP_1 | -0.0113160762092400 | <0.001 |
| abonds^2^ × Zagreb_group_index_2 | 0.0004861136272608 | <0.001 |
| Abonds × dbonds × logP_2 | -0.0927890198408315 | <0.001 |
| Abonds × Number_of_basic_groups × Geometrical_diameter | 0.1154412150487310 | 0.001 |
| Abonds × Geometrical_radius^2^ | -0.2212903922578030 | <0.001 |
| dbonds^2^ × Number_of_HBA_2 | -0.5382945677034880 | <0.001 |
| dbonds × Number_of_HBA_2 × Geometrical_radius | 1.1816430765052300 | <0.001 |
| dbonds × Fraction_of_rotatable_bonds × nOHNH | -2.1312000538909100 | 0.004 |
| dbonds × Kier_shape_2 × Zagreb_group_index_2 | -0.0049978113352559 | <0.001 |
| dbonds × Kier_shape_2 × MW | 0.0045537542244988 | <0.001 |
| TPSA × LogP_1 × Number_of_N_atoms | -0.0095294853120733 | <0.001 |
| TPSA × MW^2^ | 0.0000007966287599 | 0.001 |
| LogP_1 × Number_of_basic_groups × Geometrical_radius | 1.3378653663208400 | 0.03 |
| LogP_1 × Fraction_of_rotatable_bonds^2^ | 1.4781592851714100 | 0.002 |
| LogP_1 × Number_of_N_atoms × Geometrical_diameter | 0.0743081402575104 | <0.001 |
| Number_of_HBA_2 × Fraction_of_rotatable_bonds × Number_of_N_atoms | -2.5674022626220600 | <0.001 |
| Number_of_HBA_2 × Fraction_of_rotatable_bonds × nOHNH | 1.8073678268980800 | <0.001 |
| Number_of_HBA_2 × Number_of_Br_atoms × Kier_shape_2 | -1.9856239041564800 | <0.001 |
| Number_of_HBA_2 × Zagreb_group_index_2 × MW | 0.0000229126019259 | 0.012 |
| Number_of_basic_groups^2^ × Geometrical_radius | 63.2582529885349000 | <0.001 |
| Number_of_basic_groups × Geometrical_radius^2^ | -65.0566510398217000 | <0.001 |
| Number_of_basic_groups × logP_2^2^ | -0.8333319089644540 | 0.001 |
| Fraction_of_rotatable_bonds^2^ × Geometrical_diameter | -1.4860422088090400 | <0.001 |
| Fraction_of_rotatable_bonds × Geometrical_radius × logP_2 | 5.5945839541445500 | 0.006 |
| Fraction_of_rotatable_bonds × Zagreb_group_index_2 × logP_2 | 0.0139455085577589 | 0.001 |
| Fraction_of_rotatable_bonds × logP_2^2^ | 0.3614704478048440 | 0.001 |
| Fraction_of_rotatable_bonds × logP_2×MW | -0.0103613360461844 | 0.001 |
| Number_of_Br_atoms^2^ × Zagreb_group_index_2 | 0.0349051971614156 | 0.003 |
| Geometrical_radius^2^ × MW | -0.0286076522929991 | <0.001 |

Table S6: Descriptive stats of input data used in modelling of ODT (Equation S1).

| Variable | Mean | Minimum | Maximum |
| --- | --- | --- | --- |
| $\ln ODT$ | 2.752586 | -14.4889 | 5.283204 |
| abonds | 2.2465 | 0 | 16 |
| Aldehyde | 0.09105 | 0 | 1 |
| Carboxylic Acid | 0.03689 | 0 | 1 |
| dbonds | 0.8359 | 0 | 5 |
| Ester | 0.1476 | 0 | 2 |
| Fraction_of_rotatable_bonds | 0.29549 | 0 | 0.875 |
| Geometrical_diameter | 7.7312 | 2.0378 | 18.879 |
| Geometrical_radius | 1.0481 | 0.9456 | 1.1016 |
| Ketone | 0.1421 | 0 | 2 |
| Kier_shape_2 | 4.8349 | 0 | 19.0476 |
| LogP_1 | 4.5867 | -0.2358 | 13.551 |
| LogP_2 | 2.5693 | -2.714 | 8.732 |
| MW | 152 | 30.03 | 409.78 |
| nOHNH | 0.3909 | 0 | 6 |
| Number_of_basic_groups | 0.01648 | 0 | 1 |
| Number_of_Br_atoms | 0.01884 | 0 | 2 |
| Number_of_HBA_2 | 1.5377 | 0 | 8 |
| Number_of_N_atoms | 0.3367 | 0 | 4 |
| Sulfide | 0.1413 | 0 | 4 |
| Thiol | 0.02355 | 0 | 1 |
| TPSA | 28.117 | 0 | 157.69 |
| Vinylic Carbon | 0.6672 | 0 | 8 |
| Zagreb_group_index_2 | 111.38 | 3 | 360 |

Table S7: Comparison of model errors and ODT variability

|  | Definition | n | Mean | StDev | Min | Median | Max | IQR |
| --- | --- | --- | --- | --- | --- | --- | --- | --- |
| ODT data variance | StDev (LnODT)/ Ln(Median ODT) | 411 | 2.367 | 14.621 | 0.000 | 0.543 | 249.431 | 0.986 |
| Model variance | Model residual/ Ln(Median ODT) | 411 | 2.219 | 16.080 | 0.009 | 0.461 | 299.354 | 0.908 |


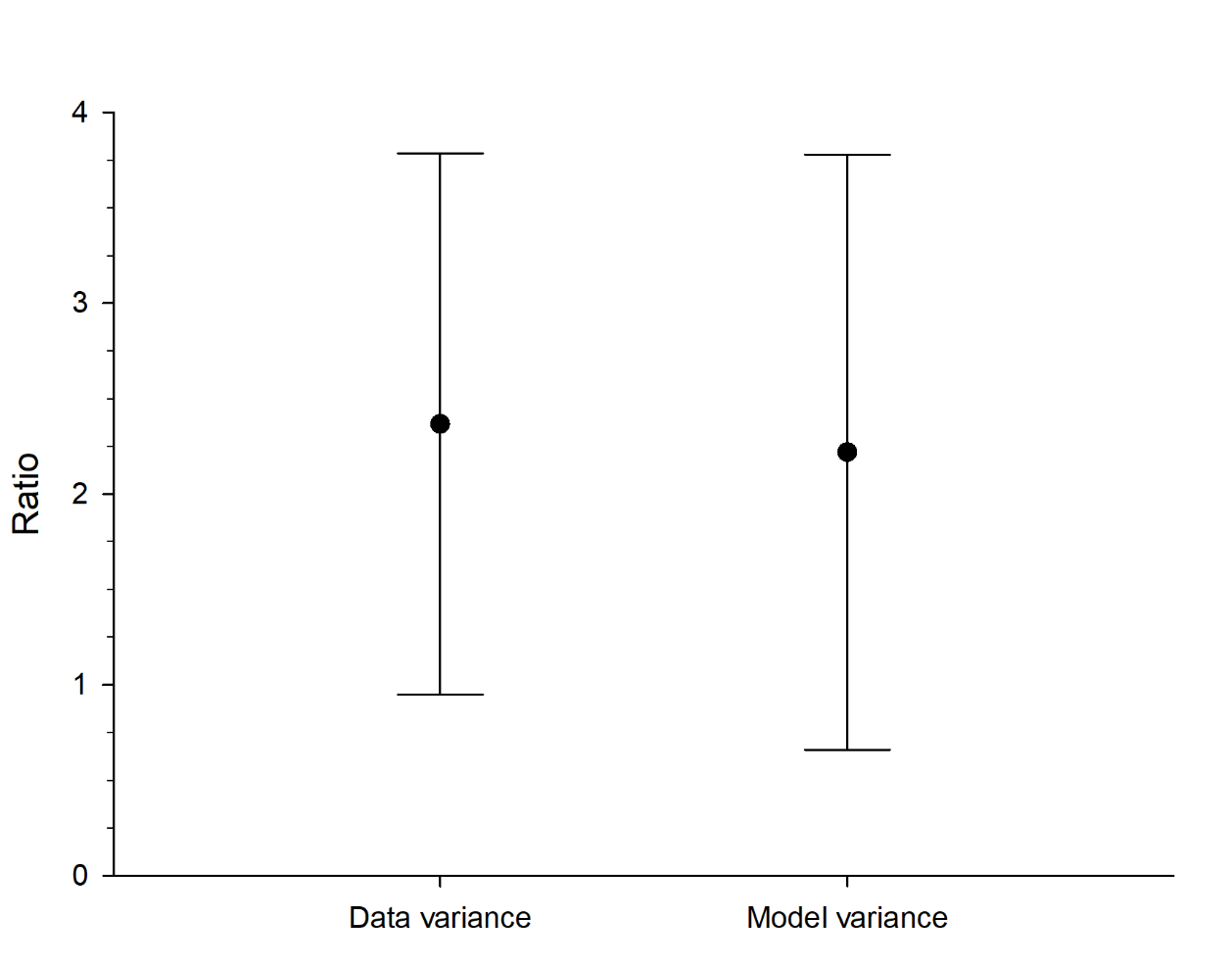


Figure S1. Comparison of variance of ODT data and model errors. Data represent means and 95% confidence interval.

Table S8: Comparison of prediction of cannabis SD from terpene or OI profiles.

|  | Proportion correctly assigned | | |
| --- | --- | --- | --- |
|  | Terpene | OI | P-value |
| Herbal | - | 0.883019 | <0.001 |
| Sweet | - | 0.781132 | <0.001 |
| Berry | 0.475472 | 0.830189 | <0.001 |
| Tree_Fruit | 0.377358 | 0.898113 | <0.001 |
| Roasted | 0.65283 | 0.85283 | <0.001 |
| Mellow | 0.396226 | 0.626415 | <0.001 |
| Mineral | 0.788679 | 0.788679 | <0.001 |
| Earthy | 0.781132 | 0.807547 | 1 |
| Tropical | 0.901887 | 0.871698 | 0.452 |
| Warming | 0.792453 | 0.8 | 0.272 |
| Citrus | 0.85283 | 0.769811 | 0.014 |
| Floral | 0.271698 | 0.233962 | 0.317 |
| Animal | - | - | - |

# Appendix 3: Equations for prediction of SD.

Equation S9: Logit function

$$P\left( 1 \right)= \frac{e^{Y'}}{(1+e^{Y^{'}})}$$

Equation S10:

$${Y'}_{Citrus}=-0.91+5.5\times beta ocimene+23.84\times linalool-941.02\times camphene\times alpha humulene+18124.51\times camphene\times trans nerolidol-580.483\times camphene \times beta caryophyllene-192.01\times alpha humulene\times linalool-17.31\times beta ocimene\times linalool-20445.4\times alpha pinene\times camphene\times terpinolene+505.60\times alpha pinene\times alpha humulene\times beta caryophyllene+68173.2\times{camphene}^{2}\times linalool+271.35\times camphene\times{beta myrcene}^{2}+6208.11\times camphene\times{terpinolene}^{2}+676.15\times beta myrcene\times alpha humulene\times linalool-331.80\times beta myrcene\times{linalool}^{2}-27.12\times beta myrcene\times{beta caryophyllene}^{2}+3580.96\times alpha humulene\times trans ocimene \times linalool+32.68\times{beta ocimene}^{2}\times terpinolene+6072.15\times beta ocimene\times trans ocimene\times trans nerolidol+353.06\times beta ocimene\times{linalool}^{2}+10510.89\times linalool\times{trans nerolidol}^{2}$$

Equation S11:

$${Y'}_{Berry}=-1.33-17.92\times alpha humulene+25.42\times{alpha pinene}^{2}-64.51\times trans ocimene\times beta caryophyllene-240.79\times{alpha pinene}^{2}\times beta limonene+367.85\times alpha pinene\times beta myrcene\times alpha humulene+31172.53\times{camphene}^{2}\times trans ocimene+65.44\times{alpha humulene}^{2}\times beta ocimene-318.99\times alpha humulene\times beta limonene\times linalool+437.37\times alpha humulene\times{linalool}^{2}+98.35\times trans ocimene\times{beta caryophyllene}^{2}$$

Equation S12:

$${Y'}_{Berry}=-1.33-200.38\times camphene-3.07\times beta ocimene\times beta caryophyllene-2941.69\times camphene\times alpha humulene\times linalool+1348.40\times camphene\times linalool\times beta caryophyllene+84700.34\times beta myrcene\times eucalyptol\times trans nerolidol$$

Equation S13:

$${Y'}_{Tree Fruit}=-1.62+3.88\times alpha pinene-159.90\times trans ocimene\times linalool+15949.24\times beta myrcene\times eucalyptol\times beta ocimene-2200.44\times trans ocimene\times trans nerolidol\times beta caryophyllene+79.86\times trans ocimene\times{beta caryophyllene}^{2}-2903.99\times beta limonene\times terpinolene\times linalool$$

Equation S14:

$${Y'}_{Earthy}=-1.28+2.23\times beta myrcene-26.70\times alpha pinene\times beta caryophyllene-19.16\times beta myrcene\times trans ocimene+323.56\times alpha pinene\times{trans ocimene}^{2}+4397.28\times{camphene}^{2}\times linalool+738.08\times alpha humulene\times terpinolene\times linalool$$

Equation S15:

$${Y'}_{Mineral}=-1.64-11.66\times camphene+1.25\times beta ocimene-2.01\times trans ocimene+38.35\times trans nerolidol-5423.51\times camphene\times trans nerolidol-21.57\times beta ocimene\times trans ocimene$$

Equation S16:

$${Y'}_{Roasted}= -1.69-2.25\times beta myrcene+50.88\times alpha pinene\times trans ocimene\times beta caryophyllene+16.64\times beta myrcene\times alpha humulene\times beta caryophyllene+114.85\times alpha humulene\times beta ocimene\times trans ocimene-37.25\times beta limonen\times linalool\times beta caryophyllene$$

Equation S17:

$${Y'}_{Mellow}=-0.90-1.40\times beta myrcene$$

Equation S18:

$${Y'}_{Warming}=-1.43+16138.43\times{camphene}^{2}\times linalool-961.59\times camphene\times beta myrcene\times linalool+2.90\times{beta myrcene}^{2}\times beta limonene-33.70\times trans ocimene\times beta limonene\times beta caryophyllene$$

Equation S19:

$${Y'}_{Floral}=-2.64+31.06\times{beta myrcene}^{2}\times trans ocimene+7187.14\times beta myrcene\times eucalyptol\times linalool-360.09\times beta myrcene\times trans ocimene\times beta limonene+703.74\times alpha humulene\times linalool\times trans nerolidol+225.85\times beta ocimene\times trans ocimene\times linalool$$

Equation S20:

$${Y'}_{{Citrus}_{SD}}=-0.35-2.26\times clove-3.03\times earthy+10.81\times bois de rose\times lemon peel+2.45\times{bois de rose}^{2}\times campherous-6.66\times citrus\times{lemon peel}^{2}+15.79\times cyrpess\times{peppery}^{2}$$

Equation S21:

$${Y'}_{Tropical}=-0.16-5.99\times blueberry+7.63\times campherous\times citrus-21.32\times citrus\times lemon peel-57.34\times clove\times peppery+9.28\times{blueberry}^{2}\times bois de rose+3.66\times bois de rose\times{pine}^{2}-7.44\times{campherous}^{2}\times pine-3.18\times campherous\times{citrus}^{2}+10.93\times campherous\times citrus\times peppery+68.53\times campherous\times clove\times peppery-45.84\times campherous\times{peppery}^{2}-2.89\times citrus\times{peppery}^{2}+13.84\times{clove}^{2}\times pine+35.87\times clove\times lemon peel\times pine+79.51\times clove\times{peppery}^{2}-10.66\times clove\times{pine}^{2}$$

Equation S22:

$${Y'}_{Berry}=-1.98+17.33\times clove\times fir needle+2.88\times earthy\times herbal-10.49\times fir needle\times lemon-27.49\times{campherous}^{2}\times spicy$$

Equation S23:

$${Y'}_{Tree Fruit}=1.74-26.55\times campherous-53.99\times dry+42.49\times{fir needle}^{2}-15.30\times{peppery}^{2}-20.10\times blueberry\times fir needle-111.12\times campherous\times fir needle+12.45\times campherous\times woody+4.64\times citrus\times clove+86.58\times clove\times fir needle+176.20\times dry\times lemon peel+56.50\times dry\times peppery-4.01\times dry\times terpenic+7.58\times fir needle\times herbal-1.72\times floral\times woody-68.38\times lemon peel\times woody+4.07\times peppery\times woody+42.04\times campherous\times earthy\times floral+45.84\times{clove}^{2}\times earthy-77.50\times{clove}^{2}\times fir needle+272.53\times clove\times earthy\times fir needle-37.55\times clove\times earthy\times floral+1.45\times clove\times{floral}^{2}-167.77\times{earthy}^{2}\times fir needle-231.14\times earthy\times{fir needle}^{2}+762.57\times earthy\times fir needle\times lemon peel-40.34\times{fir needle}^{2}\times waxy$$

Equation S24:

$${Y'}_{{Earthy}_{SD}}=-1.22+18.90\times campherous\times earthy-1.06\times citrus\times woody-31.52\times clove\times fir needle+28.71\times cypress\times peppery-77.29\times dry\times eucalyptus+57.99\times dry\times fir needle-13.13\times dry\times peppery-34.86\times earthy\times lemon peel+4.73\times green\times woody+8.60\times lemon peel\times peppery+2.73\times clove\times earthy\times woody-39.87\times{earthy}^{2}\times green$$

Equation S25:

$${Y'}_{Mineral}=-0.45-65.45\times dry\times lemon peel+19.55\times dry\times lime+24.28\times earthy\times peppery+58.40\times fir needle\times lemon peel-0.77\times green\times terpenic-5.90\times lemon\times pine-30.44\times lemon peel\times waxy+27.27\times lime\times peppery-2.74\times peppery\times woody+425.08\times clove\times cyrpress\times earthy$$

Equation S26:

$${Y'}_{Roasted}=-1.38-0.35\times citrus+4.43\times thyme$$

Equation S27:

$${Y'}_{Mellow}=-2.50+2.27\times peppery+3.46\times citrus\times clove-2.34\times clove\times woody-10.33\times{blueberry}^{3}+19.27\times campherous\times{clove}^{2}-11.42\times campherous\times dry\times woody-9.39\times citrus\times clove\times dry+0.90\times citrus\times clove\times woody-112.61\times{clove}^{2}\times cypress$$

Equation S28:

$${Y'}_{Warming}=-0.97-2.33\times waxy-8.29\times citrus\times earthy-10.33\times{clove}^{3}+15.98\times{blueberry}^{2}\times campherous+18.56\times blueberry\times clove\times dry+155.57\times blueberry\times{earthy}^{2}-20.11\times bois de rose\times campherous\times peppery+69.87\times{clove}^{2}\times cypress+42.90\times{clove}^{2}\times earthy+26.65\times{clove}^{2}\times peppery-125.63\times clove\times{earthy}^{2}-17.18\times clove\times{peppery}^{2}+20.67\times earthy\times{peppery}^{2}$$

Equation S29:

$${Y'}_{Herbal}=-1.32+14.28\times eucalyptus-1.69\times lemon-24.08\times campherous\times clove-17.33\times{clove}^{3}+0.21\times{floral}^{3}+90.84\times campherous\times clove\times earthy+99.74\times{clove}^{2}\times cypress+13.73\times{clove}^{2}\times floral-3.03\times clove\times{floral}^{2}-22.10\times{earthy}^{2}\times woody+1.80\times earthy\times{woody}^{2}$$

Equation S30:

$${Y'}_{Floral}=-2.57+8.78\times blueberry-8.48\times fir needle-4.85\times green+259.81\times earthy\times eucalyptus\times lemon peel-31.72\times eucalyptus\times green\times lemon+1.98\times fir needle\times herbal\times lemon$$

Equation S31:

$${Y'}_{Sweet}=-1.71+1.80\times tropical+3.40\times fir needle\times green-15.72\times{earthy}^{2}\times floral+442.88\times earthy\times eucalyptus\times lime+22.32\times earthy\times{fir needle}^{2}+earthy\times floral\times green-106.32\times{eucalyptus}^{2}\times herbal-368.53\times{fir needle}^{2}\times lemon peel-9.90\times fir needle\times green\times lemon+30.34\times fir needle\times herbal\times lemon peel-3.22\times herbal\times{lemon peel}^{2}$$
